# The impact of parental accompaniment in paediatric trauma: a helicopter emergency medical service (HEMS) perspective

**DOI:** 10.1186/1757-7241-22-32

**Published:** 2014-05-13

**Authors:** Alan Cowley, Neal Durge

**Affiliations:** 1 Kent, Surrey, Sussex Air Ambulance Trust Wheelbarrow Park Ind, Est. Pattenden Lane, Marden, Kent TN12 9QJ, UK; 2 South East Coast Ambulance Service NHS Foundation Trust, Banstead, UK; 3 Royal London Hospital, Whitechapel, London, UK

**Keywords:** HEMS, Parent, Accompany, Child, Trauma, Stress, Pre-hospital, Transport

## Abstract

Major trauma remains a significant cause of mortality and morbidity in young people and adolescents throughout the western world. Both the physical and psychological consequences of trauma are well documented and it is shown that peri-traumatic factors play a large part in the emotional recovery of children involved in trauma. Indeed, parental anxiety levels may play one of the biggest roles. There are no publically available guidelines on pre-hospital accompaniment, and where research has been done on parental presence it often focuses primarily on the parents or staff, rather than the child themselves. Whilst acknowledging the impact on parents and staff, the importance of the emotional wellbeing of the child should be reinforced, to reduce the likelihood of developing symptoms in keeping with post-traumatic stress disorder. This non-systematic literature review, aims to examine the impact of parental accompaniment to hospital, following paediatric trauma, and to help pre-hospital clinicians decide whether accompaniment would be of benefit to their patient population. The lack of published data does not enable a formal recommendation of parental accompaniment in the helicopter to be mandated, though it should be the preference in land based conveyance. Future research is needed into the emotional recovery of children after trauma, as well as the experiences of patient, parent and staff during conveyance.

## Introduction

Major trauma remains a significant cause of mortality and morbidity in young people and adolescents throughout the western world [1]. This article was prompted by some recent cases in which HEMS clinicians were unable to allow the parent to accompany the child to hospital in the aircraft. In one case the child went on to develop significant emotional and behavioural problems. Whilst there was no evidence that the absence of the parent during transport to hospital had any direct causation, it seems pertinent to explore the consequences of parental accompaniment and to assess the question of whether parents should routinely accompany their children to hospital, particularly when travelling by air.

We explored the impact of parental accompaniment on four main groups; the patient, the parent, the staff and the flight crew, with the patient remaining the primary focus. For ease of understanding the term ‘parent’ will be used throughout the article, though it is accepted that this may refer to any parental figure.

The consequences of trauma are well documented, but robust evidence on the subject of whether parental accompaniment improves a child’s subsequent emotional wellbeing is scarce. Several studies suggest parental anxiety plays a big role [2-5] and numerous others skirt the peripheries of the topic with Trickey et al. [2] and many others looking at PTSD risk factors in children, whilst Davies et al. [6] look at whether parents should accompany children on inter-hospital transfers, but all very much from the parent and staff member’s viewpoint. Tasker [7] provides an excellent commentary on the current standpoint in the inter-hospital environment, and highlights the confusion and inconsistencies that exist from service to service. We discuss the effect of the conveyance on the child, parent and staff member, and draw together literature from several fields including child psychology, aviation safety and critical care transfers in an attempt to provide some better defined parameters to assist the pre-hospital clinician in making the decision. We also emphasise why the decision is so important, in that the patient’s pathway does not finish when handed over to the hospital team, and that decisions made in the field can have long lasting emotional consequences.

Numbers vary, but studies suggest anywhere from 25% upwards of children involved in a road traffic collision (RTC) will develop PTSD-type symptoms [8-10]. If we can better understand the parents’ role in the development of these symptoms, and specifically if parental accompaniment to hospital can help to reduce their likelihood, then it will go some way to ensuring that the patient is receiving the best care emotionally, as well as physically.

This is our primary objective, to draw together the literature surrounding parental accompaniment, but with the patient’s wellbeing as the primary focus, rather than solely the opinion of the parent or staff member. This has the secondary aim of making recommendations to pre-hospital clinicians, primarily those involved in air transport, as to whether the parent should be allowed to travel with the patient in the majority of instances.

### Existing guidelines

There are two sets of guidelines available in the UK that cover the subject of parental accompaniment of critically ill children, though they are both designed for inter-hospital transfers rather than the pre-hospital environment, and so currently it would be left to local policy to dictate. The Paediatric Intensive Care Society [11] makes no direct referral to accompaniment but states that parents should be given all possible help regarding transport to the child’s unit or hospital. The National Coordinating Group on Paediatric Intensive Care [12] embellishes slightly, agreeing that arrangements should be made to transport the child’s immediate family. It touches on the possibility of travelling in the ambulance, but states that this is often not logistically plausible and comes nowhere near making a recommendation that this should be done wherever possible. There were no publically available guidelines on parental accompaniment in the HEMS, or non-emergency, environment. Within our HEMS unit there is no formal standard operating procedure, but an effort is made to ensure the parent will accompany wherever logistically possible.

### PTSD in children and infants

PTSD development is generally not at the forefront of the pre-hospital clinician’s mind, but children, as might be expected, react to traumatic incidents in very different ways depending upon specific individual factors as well as generic ones such as age, sex, injury severity etc. Children and post-traumatic emotional development is a complex area and not within the scope of this article to address it in detail. However, it would be remiss not to highlight its role and importance in the decision making process. It is worth noting that much of the literature distinguishes between older children (above 7–8 years) in which PTSD is well studied and defined, and younger children, in which there is no truly validated tool for PTSD diagnosis [10].

PTSD itself covers a wide range of symptoms that occur in the time following a traumatic event. The list below outlines the criteria, according to DSM-5 [13].

DSM-5 Criteria - all must be satisfied [13]

• Exposure to a traumatic event

• Persistent re-experiencing

• Persistent avoidance and emotional numbing

• Duration of symptoms for more than one month

• Significant impairment

The symptoms themselves are widespread, and categorised in a number of ways in the literature. Cohen et al. [3] succinctly breaks them in to 4 areas:

– *affective symptoms* (fear, depression, anger, mood changes)

– *behavioural* (avoidance, substance abuse, self-harm)

– *cognitive* (irrational belief, self-blame)

– *complex* (poor self-esteem, relationship breakdown, withdrawal from social circle)

However, it can be seen that PTSD symptoms are highly varied and generally occur as a continuum, ranging from an isolated minor symptom through to overwhelming symptoms causing a catastrophic breakdown of normal life [3]. It is acknowledged that exposure to trauma may also cause other symptoms, that fall short of a definitive PTSD diagnosis, but can’t be underestimated. These can include separation anxiety and regressive infantile behaviour in the very young with older children becoming irritable, angry and destructive.

### Parental effect on PTSD development in children

The reaction of the parents in trauma is a good indicator of emotional anxiety and PTSD likelihood in children, especially at younger ages [2-5,14]. The literature strongly shows the correlation between the anxiety levels of the parent, and the subsequent emotional recovery of the child. As the visible anxiety of the parent increases, it is likely to be detrimental to the child, both in the short and long term. There is some limited evidence to suggest that separation from parents during trauma can increase the likelihood of PTSD, but it appears this may need to be balanced according to the parent’s anxiety levels.

Unfortunately very few robust articles appear to directly address the issue of parental separation during trauma, and its effect on the child. Shah & Mudholkar [15] suggest that children who are injured in the absence of their parents may have an increased risk of PTSD, but this is not entirely the same point. Studies also suggest [16,17] that subsequent separation from parents can sharply increase the risk of PTSD. However, these studies are not directly applicable as they are taken from young people’s experiences of growing up in dangerous surroundings or following natural disasters, rather than an acute traumatic event, such as a road traffic accident. Trickey [2] and Bui [18] both show peri-traumatic distress to be a risk factor, but neither address parental separation specifically, whilst Lewis et al. [19] anecdotally suggest an emotional benefit of accompaniment, for both child and parent.

Looking elsewhere at the involvement of parents during other traumatic incidents within healthcare, Boudreaux et al. [20] offered some anecdotal evidence that parental presence during emergency department (ED) procedures may benefit the patient, but Chundamala et al. [21] showed no apparent benefit to parent or child when the parent was present for anaesthesia induction.

### Other factors

Aside from the parental factor, there are many other aspects of trauma that play a large part in dictating the child’s emotional response [2]. Age is not shown to be significant in PTSD, and young age does not prevent it [2]. Unsurprisingly the magnitude of the trauma, and the injury severity score (ISS) is shown to have a positive correlation with the extent of PTSD, and though amnesia offers a limited level of protection, intubation and anaesthesia are unlikely to negate the onset of PTSD in either the child or parent [22]. Pharmacological interventions are common and can be effective. Midazolam can be effective in reducing anxiety, and promoting amnesia [23]. Analgesia in general can calm the child, which is likely to ease the parent’s anxiety. However, these are not factors that will be affected by the presence, or absence, of a parent.

### Impact on staff and parents

The wish of the parent must of course be taken in to account, but it should be remembered that the decision of whether the parent travels with the child will also have an impact upon the parent, and the staff. A postal study of parents showed a high proportion wished to be involved in the transfer of their child in the inter-hospital environment [24], and subsequent policy changes within the South Thames Retrieval Service reduced parental stress as a result [6]. In the hospital environment, parents are increasingly keen to be involved with their child’s interventions, though this decreases as the intervention becomes more serious and time critical [25]. Nevertheless, in the ED parents are routinely encouraged to observe their child’s care. Results vary in the parent’s desire to be involved but surveys show that up to 94% of parents would want to accompany their child to hospital where possible [7].

Post-traumatic emotional prognosis is not a subject that should be exclusive to the patient. PTSD development, following their child’s accident, has been shown to occur in up to 20% of mothers and 12% of fathers [26]. It has also been suggested, though evidence exists only in non-traumatic disorders, that parental PTSD may subsequently affect the child’s long-term recovery [27].

Davies et al. [6] performed the most applicable survey regarding staff opinions on parental accompaniment. They found that 96-98% of staff on critical transfers perceived little or no increased stress when the parent was allowed to travel. This is an important point on the aircraft since the medical crew also perform a vital function in maintaining the integral safety of the flight. Any increased stress, above and beyond that provided by the patient, should be viewed as inherently dangerous to the flight itself. With this in mind it is worth noting that Davies *et al’s*[6] study also observed that on 4% of the journeys, staff reported an adverse event; the majority of which were due to the parent becoming unwell or aggressive. This is a critical factor when on the aircraft as the environment is a hostile one due to noise and the inability to communicate. The HEMS crew should be able to satisfy themselves that neither of these will be an issue prior to take off, if the parent is to travel.

### Aviation safety

A HEMS mission is set apart from most other critical care conveyance methods due to the presence of aviation. Aviation safety is the underpinning principle of every HEMS operation and the adage “aviate before you medicate” is well known amongst HEMS pilots and crew members alike. On a HEMS mission, the pilot is afforded several exemptions that they can use at their discretion should they feel it would benefit the mission [28], primarily involving the landing and take-off at ‘ad hoc’ sites.

The primary method to reduce risk is the involvement of the medical crew in the aviation process. The presence of a patient reduces the crew’s ability to do this and so, it follows that, the presence of a relative further prevents this. We have already discussed the stress levels of ambulance personnel when a relative is present, but it is safe to conclude that as you increase the number of non HEMS-qualified personnel in the aircraft, the risk to all increases. In addition, the allowance of a HEMS aircraft to perform a take-off and landing with increased risk is the primary reason that the crew wear aviation specific personal protective equipment (PPE)- something rarely afforded to patients or relatives for logistical reasons. Therefore the risk to their health, should an accident occur, is greatly increased also.

### The hospital experience

The patient’s journey does not stop once handed over to the hospital. Although the pre-hospital team can have little effect on the actions of the hospital, whether positive or negative, it should be remembered that if the parents have not accompanied the patient, then they will be without them, at least for the first part of their hospital experience. This can often be even more stressful than the pre-hospital environment, with increased numbers of personnel, machines, patient movements and potentially invasive procedures and treatments. We have seen that several studies show peri-traumatic stress to be a good indicator for PTSD, and this hospital experience should be seen as a continuation of the traumatic experience. McFarlane [4] and Kolaitis et al. [17] state that separation from parents in the time following a traumatic event were powerful determinants of post-traumatic phenomena.

## Discussion

We have emphasised that during a HEMS mission, aviation safety must remain of paramount importance and there is no doubt that by increasing the number of non-HEMS trained personnel on an aircraft, safety is compromised to some degree. Nonetheless, the long term emotional wellbeing of the patient and parent can be directly affected at this point and need to be carefully considered.

It is widely acknowledged that the mental wellbeing of the child is very much related to the anxiety levels of the parent. A parent with high levels of anxiety can be detrimental, in both short and long term, to the patient as well as a potential source of compromised flight safety. It may be beneficial to explain this to them, rather than an outright refusal.

There is little evidence to show the benefit of a parent’s presence to the child during, or immediately after, trauma unless it can reduce peri-traumatic distress. It may be that parent and child will reduce their risk of PTSD, but further research is needed. The direct impact of an accompanying parent on the staff appears to be negligible, though even a small risk is highly significant in the aircraft. Considering this, albeit small, increased aviation risk, it is not possible to formally recommend mandated parental accompaniment on the helicopter, and pending further research it should continue to be made on a case by case basis and according to local protocol. This subjective assessment should include, but not be limited to, the patient’s and parent’s emotional state, degree of injury and aviation safety, distances involved, other means of transport available and ability to use those forms of transport, in an attempt to facilitate a safe parental accompaniment.

It remains the position of our service that the clinician should err on the side of allowing the parent to travel as it appears, albeit anecdotally, that in the long run it should be beneficial for both patient and parent. This takes in to account not just the flight, but the following hospital experience as well. It is not possible to assign upper and lower age limits to this recommendation.

Where air conveyance is not applicable, either in the non-HEMS pre-hospital environment or for logistical reasons on a HEMS mission, the balance tips very much towards allowing parental accompaniment. As well as the likely benefit for patient and parent, studies show only a limited impact on the stress levels of the staff and where flight safety is now of no concern, the risks of parental accompaniment are outweighed by the potential benefits.

The pre-hospital clinician is served well by a reminder of the importance of the patient journey, and that whilst immediate medical care is a priority, consideration must also be made for the emotional wellbeing of both child and parent. Reducing parental anxiety will have a positive effect on both parties, which will reduce PTSD likelihood in the future, and so consideration should be given to this whilst on scene. Whilst the evidence is not sufficient to allow a formal recommendation in the HEMS environment, it highlights the factors that the crew need to consider in order to make an informed decision.

There is a need for future pre-hospital research, both in and out of the HEMS environment, to try to measure the psychological impact of parental accompaniment and behaviour in paediatric trauma. Future research would also need to demarcate the upper and lower ages at which parental accompaniment has a positive effect. This would undoubtedly need multidisciplinary coordination, including follow ups in the short and long term following intervention.

### Limitations

We accept this review is limited by the wide variety of subjects that must be considered when addressing such a problem. The subject of PTSD is vast and widely researched and so making definitive conclusions about the impact of parental accompaniment is precarious.

## Competing interests

The authors declare that they have no competing interests.

## Authors’ contributions

AC conceived the review, and participated fully in its research, construction and editing. ND provided expert opinion, guidance and participated fully in the drafting and editing process. Both authors read and approved the final manuscript.

## Authors’ information

Alan Cowley MCPara, BSc, MSc is a paramedic with the Kent, Surrey, Sussex Air Ambulance Trust in the UK and a paramedic and practice placement educator with South East Coast Ambulance Service NHS Foundation Trust.

Dr. Neal Durge MBBS, FCEM is a consultant in Emergency Medicine based at the Royal London Hospital and also a clinical governance lead with Kent, Surrey, Sussex Air Ambulance Trust.
